# Supplementary material for: AlphaFold predictions are valuable hypotheses and accelerate but do not replace experimental structure determination
Source: Nat Methods. 2023 Nov 30;21(1):110–6. doi: 10.1038/s41592-023-02087-4 (PMC10776388; doi:10.1038/s41592-023-02087-4)
Supplement: Supplementary file 1 — Reporting Summary [file 41592_2023_2087_MOESM1_ESM.pdf]

nature research

Corresponding author(s): Terwilliger, Thomas C

Last updated by author(s): Aug 28, 2023

Reporting Summary

Nature Research wishes to improve the reproducibility of the work that we publish. This form provides structure for consistency and transparency in reporting. For further information on Nature Research policies, see our Editorial Policies and the Editorial Policy Checklist.

Statistics

For all statistical analyses, confirm that the following items are present in the figure legend, table legend, main text, or Methods section.

n/a	Confirmed	
☒	☐	The exact sample size (*n*) for each experimental group/condition, given as a discrete number and unit of measurement
☒	☐	A statement on whether measurements were taken from distinct samples or whether the same sample was measured repeatedly
☐	☒	The statistical test(s) used AND whether they are one- or two-sided<br>*Only common tests should be described solely by name; describe more complex techniques in the Methods section.*
☒	☐	A description of all covariates tested
☒	☐	A description of any assumptions or corrections, such as tests of normality and adjustment for multiple comparisons
☒	☐	A full description of the statistical parameters including central tendency (e.g. means) or other basic estimates (e.g. regression coefficient) AND variation (e.g. standard deviation) or associated estimates of uncertainty (e.g. confidence intervals)
☐	☒	For null hypothesis testing, the test statistic (e.g. F, t, r) with confidence intervals, effect sizes, degrees of freedom and P value noted<br>*Give P values as exact values whenever suitable.*
☒	☐	For Bayesian analysis, information on the choice of priors and Markov chain Monte Carlo settings
☒	☐	For hierarchical and complex designs, identification of the appropriate level for tests and full reporting of outcomes
☒	☐	Estimates of effect sizes (e.g. Cohen's d, Pearson's r), indicating how they were calculated

Our web collection on statistics for biologists contains articles on many of the points above.

Software and code

Policy information about availability of computer code

Data collection	No data collection software was used.
Data analysis	Phenix software, . Software, with documentation, instructions, test data, tests, example datasets, and tutorials is available at www.phenix-online.org. Version 1.21 of Phenix (this applies to all Phenix software including phenix.refine) was used. No custom modifications were made for this study. Scripts and data for all analyses are available at: https://phenix-online.org/phenix_data/terwilliger/alphafold_crystallography_2022/)<br>ChimeraX. Version 1.2.5 was used for display of models and density maps.

For manuscripts utilizing custom algorithms or software that are central to the research but not yet described in published literature, software must be made available to editors and reviewers. We strongly encourage code deposition in a community repository (e.g. GitHub). See the Nature Research guidelines for submitting code & software for further information.

Data

Policy information about availability of data

All manuscripts must include a data availability statement. This statement should provide the following information, where applicable:

- Accession codes, unique identifiers, or web links for publicly available datasets
- A list of figures that have associated raw data
- A description of any restrictions on data availability

Data and materials availability: Input data for deposited models were taken from the Protein Data Bank. The 102 accession codes used were: 7e0m, 7fhr, 7v6p, 7Ljh, 7p3a, 7v38, 7v3b, 7o9p, 7rLz, 7qdv, 7ewj, 7rw4, 7waa, 7kdx, 7fiu, 7n3v, 7ptb, 7dtr, 7aoj, 7rc2, 7tcr, 7wja, 7vnx, 7x8v, 7raw, 7rpy, 7aov, 7tb5, 7t8L, 7vwk, 7ne9, 7nqd, 7s5L, 7wbk, 7x77, 7e3z, 7f0o, 7v1q, 7etx, 7ety, 7ecd, 7dxn, 7eyj, 7e4d, 7wsj, 7fi3, 7wnn, 7vgm, 7eio, 7v9n, 7tvc, 7Lbk, 7e6v, 7b3n, 7bLL, 7djj, 7dms, 7dqx, 7drh, 7dri, 7e1d, 7e85, 7edc, 7ejg, 7es4, 7esi, 7eus, 7ew8, 7exx, 7f2a, 7fjg, 7kzh, 7Lsv, 7mku, 7naz, 7ncy, 7nxg, 7o51, 7o5y, 7oc3, 7oom, 7oq6, 7qs4, 7rm7, 7t7j,

7tbs, 7tem, 7tfq, 7tj1, 7tL5, 7tmu, 7tog, 7toj, 7trv, 7trw, 7tt9, 7twc, 7tzp, 7unn, 7w3s, 7wdq, 8cuk. All models are downloadable from the PDB with links such as: https://files.rcsb.org/download/7tzp.pdb or (for larger models that are not available in this format) https://files.rcsb.org/download/7tzp.cif . We used the Phenix tool fetch_pdb to download models and crystallographic data for each structure. Predicted models, rebuilt models, and density-modified map coefficients are available at: https://phenix-online.org/phenix_data/terwilliger/alphafold_crystallography_2022/ along with a spreadsheet that contains all the raw data and analyses described in our previous work28 and described here. The directory terwilliger/alphafold_crystallography_2022/ contains a README file describing the contents of the site, the spreadsheet, and a data/ directory with one compressed archive for each structure containing models and crystallographic data files. This directory also contains a compressed archive (alphafold_crystallography.tgz) containing all the data and all the scripts used to create the spreadsheet.

Code Availability: All code for the Phenix version of the AlphaFold2 Colab is freely available on GitHub at https://github.com/phenix-project/Colabs. All code for Phenix is available at phenix-online.org.

Field-specific reporting

Please select the one below that is the best fit for your research. If you are not sure, read the appropriate sections before making your selection.

☒ Life sciences ☐ Behavioural & social sciences ☐ Ecological, evolutionary & environmental sciences

For a reference copy of the document with all sections, see nature.com/documents/nr-reporting-summary-flat.pdf

Life sciences study design

All studies must disclose on these points even when the disclosure is negative.

Sample size	We used all the structures from https://www.biorxiv.org/content/10.1101/2022.11.18.517112v1 yielding a free R value of 0.30 or lower (102 structures) to ensure that the density-modified electron density maps used as a reference were of high quality.
Data exclusions	None
Replication	One complete replication of all the analyses was carried out, yielding essentially identical results (mean absolute value of changes in map correlation from one replicate to the other for 208 datasets where a solution was found of 0.025).
Randomization	We did not carry out randomization because our sampling procedure (taking all the unique deposits within a time frame) provides an unbiased sampling. We controlled covariates by selecting unique deposits (removing samples that had the same sequences).
Blinding	The work was not blinded. This is a limitation of the analysis. The software was developed at the same time as the analysis and used some of the samples in the analysis in development. Consequently, it is possible that even though the same code and parameters are used for all the work shown here there may be choices made in parameters that improved results for these cases but that might not improve them for a completely new set of structures.

Reporting for specific materials, systems and methods

We require information from authors about some types of materials, experimental systems and methods used in many studies. Here, indicate whether each material, system or method listed is relevant to your study. If you are not sure if a list item applies to your research, read the appropriate section before selecting a response.

Materials & experimental systems

n/a	Involved in the study	
☒	☐	Antibodies
☒	☐	Eukaryotic cell lines
☒	☐	Palaeontology and archaeology
☒	☐	Animals and other organisms
☒	☐	Human research participants
☒	☐	Clinical data
☒	☐	Dual use research of concern

Methods

n/a	Involved in the study	
☒	☐	ChIP-seq
☒	☐	Flow cytometry
☒	☐	MRI-based neuroimaging
